# Somatic musculature in trematode hermaphroditic generation

**DOI:** 10.1186/s12862-015-0468-0

**Published:** 2015-09-15

**Authors:** Darya Y. Krupenko, Andrej A. Dobrovolskij

**Affiliations:** Department of Invertebrate Zoology, Saint Petersburg State University, Universitetskaya nab. 7/9, 199034 St. Petersburg, Russia; Department of Zoology, Herzen State Pedagogical University, St. Petersburg, Russia

## Abstract

**Background:**

The somatic musculature in trematode hermaphroditic generation (cercariae, metacercariae and adult) is presumed to comprise uniform layers of circular, longitudinal and diagonal muscle fibers of the body wall, and internal dorsoventral muscle fibers. Meanwhile, specific data are few, and there has been no analysis taking the trunk axial differentiation and regionalization into account. Yet presence of the ventral sucker (= acetabulum) morphologically divides the digenean trunk into two regions: preacetabular and postacetabular. The functional differentiation of these two regions is already evident in the nervous system organization, and the goal of our research was to investigate the somatic musculature from the same point of view.

**Results:**

Somatic musculature of ten trematode species was studied with use of fluorescent-labelled phalloidin and confocal microscopy. The body wall of examined species included three main muscle layers (of circular, longitudinal and diagonal fibers), and most of the species had them distinctly better developed in the preacetabuler region. In majority of the species several (up to seven) additional groups of muscle fibers were found within the body wall. Among them the anterioradial, posterioradial, anteriolateral muscle fibers, and U-shaped muscle sets were most abundant. These groups were located on the ventral surface, and associated with the ventral sucker. The additional internal musculature was quite diverse as well, and included up to twelve separate groups of muscle fibers or bundles in one species. The most dense additional bundles were found in the preacetabular region and were connected with the suckers.

**Conclusions:**

Previously unknown additional somatic musculature probably provides the diverse movements of the preacetabular region, ventral sucker, and oral sucker (or anterior organ). Several additional muscle groups of the body wall (anterioradial, posterioradial, anteriolateral fibers and U-shaped sets) are proposed to be included into the musculature ground pattern of trematode hermaphroditic generation. This pattern is thought to be determined by the primary trunk morphofunctional differentiation into the preacetabular and the postacetabular regions.

## Background

The flatworm somatic musculature for a long time has been regarded as one of the most simple within Metazoa. According to the classical descriptions the body wall (or *Hautmuskelschlauch*) usually comprises the circular, diagonal and longitudinal muscle fibers, and the internal (or parenchymal) musculature is mostly composed of dorsoventral muscle fibers [1, 2]. It was supposed that the order of the body-wall muscle layers may vary, the diagonal fibers may be absent, or some layers may duplicate [3], but the uniformity of the muscular pattern across the body was not a question. However data obtained in the last twenty years by means of the confocal laser scanning microscopy showed that the turbellarian muscle system is much more complex than ever described and expected [4–13]. Only in Catenulida and some Acoela a simple grid of circular and longitudinal muscle fibers was confirmed [6]. The most curious patterns of the body-wall musculature were found in many Acoela. They include several groups of muscle fibers which had not been described for the flatworms earlier, e.g. the U-shaped and cross-over [5–7]. Among non-neodermatan Rhabditophora some species have plain musculature patterns in the body wall [14, 15]; others, however, do not fit into the classical schemes either [4, 10].

Trematoda Rudolphi, 1808 (*sensu* Digenea Carus, 1863) is one of the major groups within parasitic flatworms (Neodermata). Its peculiar feature is complex life-cycle in form of heterogony – the obligate alteration of parthenogenetic and hermaphroditic generations [16]. Two larval stages are present in typical development of hermaphroditic generation: cercaria and metacercaria.

For the analysis of muscle system in trematode hermaphroditic generation we must take into account the axial body differentiation. The first ontogenetic milestone of this differentiation is the formation of highly autonomous (both in morphology and function) locomotory appendage – the tail – which will not be discussed in this paper. The second milestone is the formation of the ventral sucker. This leads to the primary trunk differentiation into two regions: the preacetabular and the postacetabular – anterior and posterior to the ventral sucker respectively [16, 17]. Pyotr Oshmarin in 1958 [18] proposed the functional difference between the two regions in adult worms. The preacetabular region is used for locomotion, and hence is expected to have prominent neuromusculature. The postacetabular region is specialized for reproduction and usually faintly contractive and less sensitive. This idea was supported by later investigations on the trematode nervous system which showed significant tapering of longitudinal nerve cords and absence of transverse commissures in the postacetabular region [19, 20]. But the traditional concept of the muscle system organization still has not changed. There were a few proper investigations on trematodes, but they mostly analyzed such highly secondary modified forms as adults of Strigeidae, Schistosomatidae, Bucephalidae, etc. [21–25]. There is a number of papers describing less modified species from diverse trematode taxa, and different ontogenetic stages [20, 26–29]. However these papers lack details.

We believe that careful study of various typical forms and early ontogenetic stages would be helpful to determine general musculature pattern in trematode hermaphroditic generation. In this study the preference was given to cercariae as they usually demonstrate less secondary modifications in general morphology (body construction) than the adult worms which may be strongly specialized (e.g. in Strigeidae, Sanguinicolidae, Heterophyidae, Renicolidae). Eight of ten studied species were represented by the stage of cercaria, and two by metacercaria (Table 1). Three of the studied species (*Sanguinicola* sp., *Cryptocotyle lingua* and *Microphallus claviformis*) have highly juvenilized cercariae which lack ventral sucker. Ten studied species belong to ten families from distant high-level taxa: Xiphidiata, Diplostomata, Echinostomata, Opisthorchiata and Bucephalata (naming after [30]). The study was carried out with use of fluorescent-labelled phalloidin staining and confocal microscopy. We report great variety of additional body-wall and internal musculature, mostly associated with the ventral sucker and the preacetabular region. Within this variety several muscular groups were recurrent among the studied species, and we consider these to be peculiar features of muscular pattern in the trematode hermaphroditic generation. Also we discuss the impact of axial differentiation and regionalization, and other alterations of the body construction on the organization of muscle system, in case of both trematodes and other flatworms.

**Table 1:** List of species studied

Family	Species	Stage	Number of specimens studied	Host
Strigeidae	*Cotylurus cornutus* (Rudolphi 1809)	Cerc	16	*Lymnaea* sp.
Sanguinicolidae	*Sanguinicola* sp.	Cerc	9	*Lithoglyphus naticoides*
Fellodistomatidae	*Fellodistomum fellis* (Olsson 1868)	Cerc	7	*Ennucula tenuis*
Gymnophallidae	*Gymnophallus* sp.	Mc	11	*Turtonia minuta*
Echinostomatidae	*Himasthla elongata* (Mehlis 1831)	Cerc	16	*Littorina littorea*
Heterophyidae	*Cryptocotyle lingua* (Creplin 1825)	Cerc	18	*Littorina littorea*
Acanthocolpidae	*Neophasis lageniformis* (Lebour 1910)	Mc	9	*Buccinum undatum*
Renicolidae	*Cercaria parvicaudata* Stunkard and Shaw 1931	Cerc	11	*Littorina saxatilis*
Lecithodendriidae	*Cercaria edgesii* Schenkov 2013	Cerc	10	*Bithynia tentaculata*
Microphallidae	*Microphallus claviformis* (Brandes 1888)	Cerc	8	*Hydrobia ulvae*

*Cerc* cercariae, *Mc* metacercariae

## Results

### Body-wall musculature

**Fig. 1:**
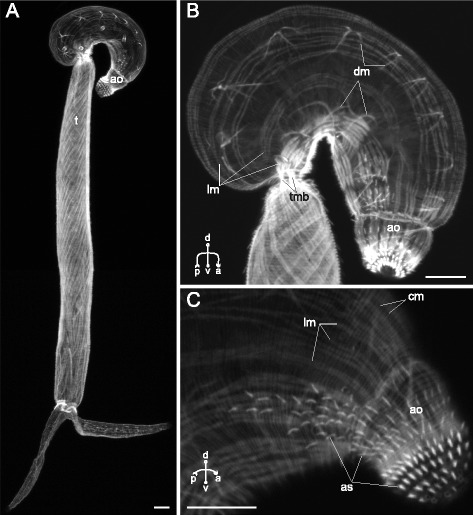
*Sanguinicola* sp. cercariae, body-wall musculature. **a**: general side view; **b**: trunk side view; **c**: side view of the anterior region. ao – anterior organ; as – actinous spines in tegument; cm – circular muscle fibers; dm – diagonal muscle fibers; lm – longitudinal muscle fibers; t – tail; tmb – dense muscle bundles close to the tail basis. Scale bars 10 μm

**Fig. 2:**
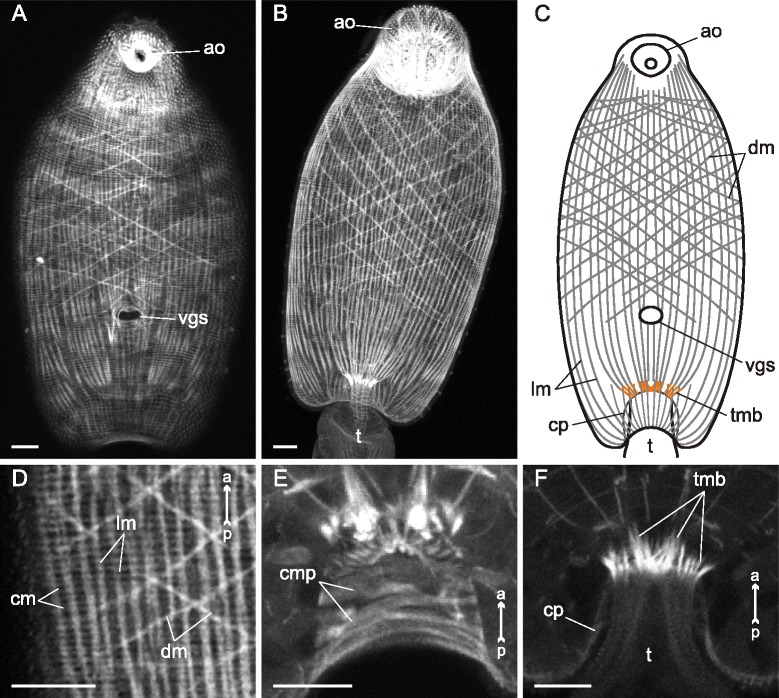
*Cryptocotyle lingua* cercariae, body-wall musculature. **a**: trunk ventral view (tail detached); **b**: trunk dorsal view; **c**: scheme showing the arrangement of longitudinal and diagonal muscle fibers on the ventral side of the trunk; **d**: part of dorsal body wall showing three main muscle layers; **e**: Z-stack of caudal pocket wall (tail detached); **f**: frontal optical section of the tail basis. ao – anterior organ; cm – circular muscle fibers; cmp – circular muscle bundles within the wall of caudal pocket; cp – wall of caudal pocket; dm – diagonal muscle fibers; lm – longitudinal muscle fibers; t – tail; tmb – dense muscle bundles close to the tail basis; vgs – ventro-genital sac primordium. Scale bars 10 μm

**Fig. 3:**
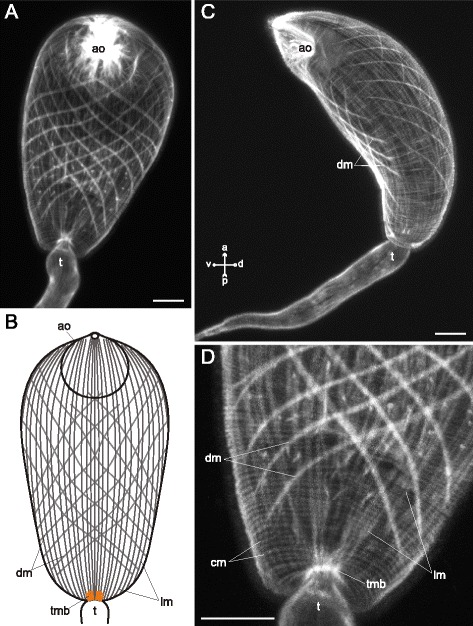
*Microphallus claviformis* cercariae, body-wall musculature. **a**: ventral view; **b**: scheme showing the arrangement of longitudinal and diagonal muscle fibers on the ventral side of the trunk; **с**: lateral view; **d**: hind part of the trunk (ventral). ao – anterior organ; cm – circular muscle fibers; dm – diagonal muscle fibers; lm – longitudinal muscle fibers; t – tail; tmb – dense muscle bundles near the tail basis. Scale bars 10 μm

The body-wall musculature of three examined species without ventral sucker (*Sanguinicola* sp., *Cryptocotyle lingua* and *Microphallus claviformis*) was an array of outer circular, intermediate longitudinal and inner diagonal muscle fibers (cm, lm and dm on Figs. 1, 2, 3 and thereafter). The circular muscle fibers did not form bundles and were compactly arranged and regularly spaced. The longitudinal muscle fibers were mostly joined into the wide bands (Figs. 1b, 2a, 3a, c). Both these layers were arranged quite uniformly along the whole trunk, but in the hind region the longitudinal fibers formed short dense bundles close to the tail base (tmb on Figs. 1b, 2c, f, 3d). *C. lingua* cercariae have deep caudal pocket, and the dense longitudinal bundles lay anterior to it and passed through the trunk to reach the tail basis (Fig. 2c, f). *C. lingua* also had thinner and rarely spaced longitudinal muscle fibers in median area of the trunk posterior region (Fig. 2a, b). The wall of the caudal pocket had exclusively circular muscle fibers forming dense irregular bands (cmp on Fig. 2e).

The diagonal muscle fibers were scarce and wider spaced than the circular and the longitudinal ones in all three species. In *Sanguinicola* sp. the layer of diagonal muscle fibers was extremely weak and uniform along the trunk (Fig. 1b). On the contrary *C. lingua* had diagonal fibers only anterior to the ventro-genital sac primordium (Fig. 2a, b), and in *M. claviformis* just few diagonal muscle fibers reached last quarter of the trunk (Fig. 3). In all three species sets of dorsal and ventral diagonal muscle fibers were clearly separated (Figs. 1b, 3c). And the diagonal muscle fibers of *Sanguinicola* sp., unlike two other species, were located rather deep beneath the longitudinal.

Other examined species had well-developed ventral sucker. They also possessed a number of specific features and additional groups of muscle fibers within the body wall. In some cases musculature differed significantly between the precetabular and the postacetabular regions. The main muscle layers of the body wall were all the same: circular, longitudinal and diagonal.

**Fig. 4:**
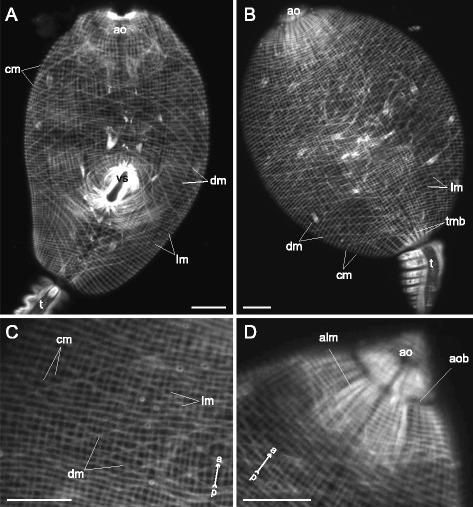
*Cercaria edgesii* cercariae, body-wall musculature. **a**: ventral view; **b**: dorsal view; **c**: part of dorsal body wall showing three main muscle layers; **d**: anterior part of the trunk (dorsal view). ao – anterior organ; alm – dense longitudinal bundles close to the anterior organ; aob – border of the anterior organ; cm – circular muscle fibers; dm – diagonal muscle fibers; lm – longitudinal muscle fibers; t – tail; tmb – dense muscle bundles close to the tail basis; vs – ventral sucker. Scale bars 10 μm

**Fig. 5:**
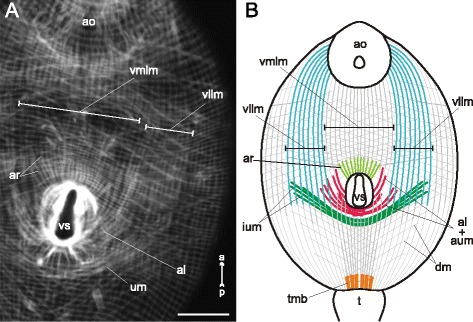
*Cercaria edgesii* cercariae, body-wall musculature. **a**: part of the trunk (ventral view); **b**: scheme showing the arrangement of longitudinal, diagonal, and additional groups of muscle fibers on the ventral side of the trunk. al + aum – anteriolateral muscle fibers with aU-shaped muscle set; ao – anterior organ; ar – anterioradial muscle fibers; dm – diagonal muscle fibers; ium – iU-shaped muscle set; t – tail; tmb – dense muscle bundles close to the tail basis; vllm – ventrolateral longitudinal muscle bands; vmlm – ventromedial longitudinal muscle fibers; vs – ventral sucker. Scale bar 10 μm

*Cercaria edgesii* (Figs. 4, 5) possessed the most weakly developed ventral sucker among these species. The layer of circular muscle fibers was uniform along the whole trunk; these fibers were regularly spaced and did not form bundles. The longitudinal muscle fibers generally did not form bundles or bands either, except for three areas: (1) thick bundles near the tail basis (tmb on Fig. 4b), (2) the medial area close to the anterior organ on the dorsal side (alm on Fig. 4d), and (3) the ventrolateral bands in the preacetabular region (vllm on Fig. 5a, b). The diagonal muscle fibers were present in both pre- and postacetabular regions, though they were more widely spaced in the hinder areas of the trunk (Fig. 4a, b). Dorsal and ventral sets of the diagonal fibers were more clearly separated in the postacetabular region.

Three additional groups of muscle fibers were located near the ventral sucker opening of *Cercaria edgesii*. The first (anterioradial) group consisted of short thin fibers radiating from the anterior border of the ventral sucker (ar on Fig. 5a, b). Within the second (anteriolateral) group the thicker muscle fibers proceeded anteriolaterally from the lateral borders of the ventral sucker. Their posterior ends were attached either near the lateral borders of the sucker or posterior to the sucker opening. Thus the part of the anteriolateral muscle fibers formed an arch termed here as the aU-shaped muscle set (“a” corresponds to “anteriolateral”) (al + aum on Fig. 5a, b). The third additional group located posterior to the aU-shaped set was a wider arch of dense muscle fibers – iU-shaped set (“i” stands for “independent”) (ium on Fig. 5a, b).

**Fig. 6:**
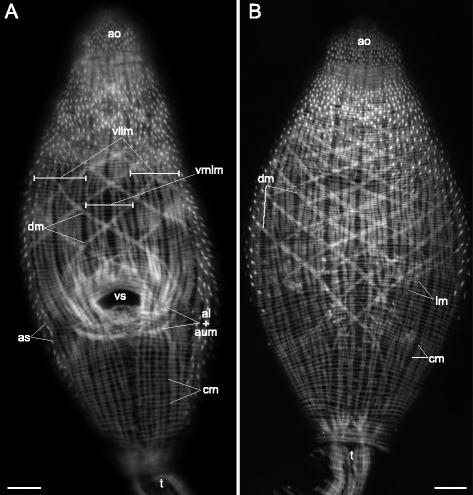
*Cotylurus cornutus* cercariae, body-wall musculature. **a**: trunk ventral view; **b**: trunk dorsal view. al + aum – anteriolateral muscle fibers with aU-shaped muscle set; ao – anterior organ; as – actinous spines in tegument; cm – circular muscle fibers; dm – diagonal muscle fibers; lm – longitudinal muscle fibers; t – tail; vllm – ventrolateral longitudinal muscle bands; vmlm – ventromedial longitudinal muscle fibers; vs – ventral sucker. Scale bars 10 μm

**Fig. 7:**
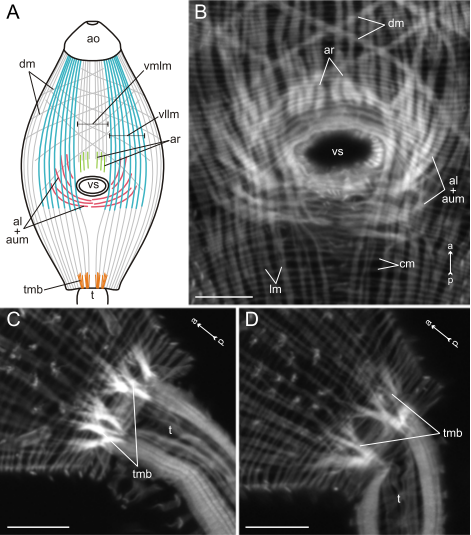
*Cotylurus cornutus* cercariae, body-wall musculature. **a**: scheme showing the arrangement of longitudinal, diagonal, and additional groups of muscle fibers on the ventral side of the trunk; **b**: arrangement of body-wall musculature around the ventral sucker opening; **c**: tail basis, dorsal view; **d**: tail basis, ventral view. al + aum – anteriolateral muscle fibers with aU-shaped muscle set; ao – anterior organ; ar – anterioradial muscle fibers; cm – circular muscle fibers; dm – diagonal muscle fibers; lm – longitudinal muscle fibers; t – tail; tmb – dense muscle bundles close to the tail basis; vllm – ventrolateral longitudinal muscle bands; vmlm – ventromedial longitudinal muscle fibers; vs – ventral sucker. Scale bars 10 μm

*Cotylurus cornutus* cercariae (Figs. 6, 7) had regularly spaced circular muscle fibers which slightly rarefied towards the posterior end of the trunk. The longitudinal muscle fibers formed wide bands in the preacetabular region, and in the postacetabular region they were joined into small bundles (2–3 fibers in each). The most dense longitudinal bands of the preacetabular region were located in the ventrolateral areas (vllm on Figs. 6a, 7a). Also thick short bundles were present near the tail basis (tmb on Fig. 7c, d). Widely-spaced diagonal muscle bundles were present only in the preacetabular region where they formed distinctly separated dorsal and ventral sets (Fig. 6a, b). Two additional groups of muscle fibers were found close to the ventral sucker opening. The first was a small group of short dense anterioradial muscle fibers (ar on Fig. 7a, b) which interdigitated with the longitudinal muscle fibers. The second group comprised dense anteriolateral muscle fibers forming aU-shaped set the same way as in *Cercaria edgesii* (al + aum on Figs. 6a, 7a, b).

**Fig. 8:**
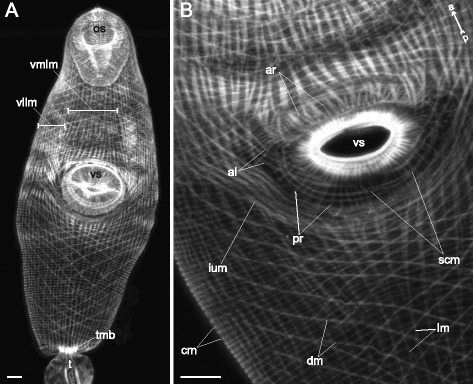
*Cercaria parvicaudata* cercariae, body-wall musculature. **a**: trunk ventral view; **b**: arrangement of muscle fibers around the ventral sucker opening. al – anteriolateral muscle fibers; ar – anterioradial muscle fibers; cm – circular muscle fibers; dm – diagonal muscle fibers; ium – iU-shaped muscle set; lm – longitudinal muscle fibers; os – oral sucker; pr – posterioradial muscle fibers; scm – semicircular muscle fibers; t – tail; tmb – dense muscle bundles close to the tail basis; vllm – ventrolateral longitudinal muscle bands; vmlm – ventromedial longitudinal muscle fibers; vs – ventral sucker. Scale bars 10 μm

**Fig. 9:**
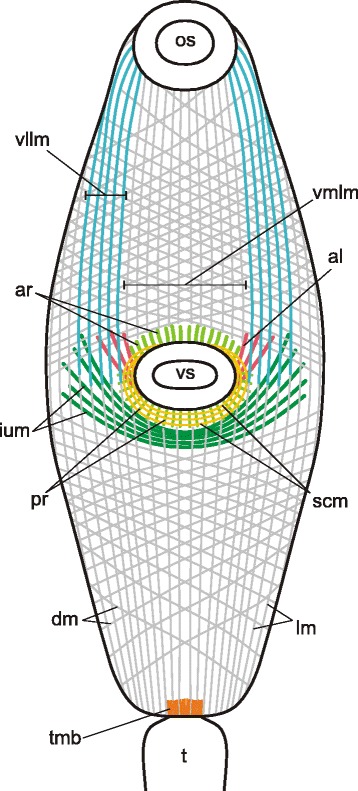
*Cercaria parvicaudata* cercariae, body-wall musculature. Scheme showing the arrangement of longitudinal, diagonal and additional muscle fibers on the ventral side. al – anteriolateral muscle fibers; ar – anterioradial muscle fibers; dm – diagonal muscle fibers; ium – iU-shaped muscle set; lm – longitudinal muscle fibers; os – oral sucker; pr – posterioradial muscle fibers; scm – semicircular muscle fibers; t – tail; tmb – dense muscle bundles close to the tail basis; vllm – ventrolateral longitudinal muscle bands; vmlm – ventromedial longitudinal muscle fibers; vs – ventral sucker

Three main muscle layers were present along the whole trunk of *Cercaria parvicaudata* though in the postacetabular region each of them was clearly wider spaced (Figs. 8, 9). The longitudinal muscle fibers were joined into small bundles that were closer packed in the ventrolateral areas of the preacetabular region (vllm on Figs. 8a, 9). Short dense bundles were present near the tail basis (tmb on Fig. 8a). Five additional groups of muscle fibers were found within the body wall. The short anterioradial muscle fibers lay close to the anterior border of the ventral sucker (ar on Figs. 8b, 9). Thinner and longer muscle fibers were radiating from the posterior and lateral borders of the ventral sucker opening, so these were termed posterioradial (pr on Figs. 8b, 9). Rare anteriolateral muscle fibers were present (al on Figs. 8b, 9). Unlike in *Cercaria edgesii*, they did not form the aU-shaped set. The iU-shaped set was well developed (ium on Figs. 8b, 9). And also a group of thin semicircular muscle fibers lay around the lateral and posterior borders of the ventral sucker (scm on Figs. 8b, 9).

**Fig. 10:**
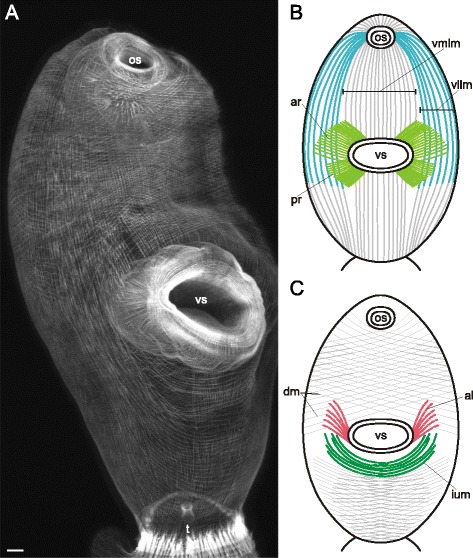
*Fellodistomum fellis* cercariae, body-wall musculature. **a**: general view of the trunk; **b**: scheme showing the arrangement of longitudinal and some of additional groups of muscle fibers; **c**: scheme showing the arrangement of diagonal and the rest of additional groups of muscle fibers. al – anteriolateral muscle fibers; ar – anterioradial muscle fibers; dm – diagonal muscle fibers; ium – iU-shaped muscle set; os – oral sucker; pr – posterioradial muscle fibers; t – tail; vllm – ventrolateral longitudinal muscle bands; vmlm – ventromedial longitudinal muscle fibers; vs – ventral sucker. Scale bar 10 μm

**Fig. 11:**
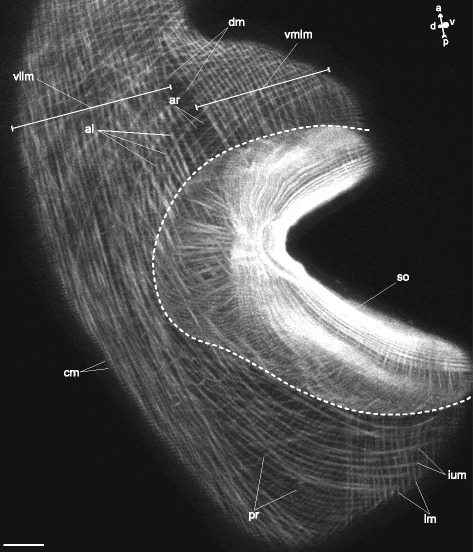
*Fellodistomum fellis* cercariae, body-wall musculature. Z-stack of oblique longitudinal optical slices to the left of the ventral sucker opening. The ventral sucker can be seen through the body wall, its border is outlined with broken line. al – anteriolateral muscle fibers; ar – anterioradial muscle fibers; cm – circular muscle fibers; dm – diagonal muscle fibers; ium – muscle fibers of iU-shaped set; lm – longitudinal muscle fibers; pr – posterioradial muscle fibers; so – ventral sucker opening; vllm – ventrolateral longitudinal muscle bands; vmlm – ventromedial longitudinal muscle fibers. Scale bar 10 μm

The body-wall musculature of the large *Fellodistomum fellis* cercariae (Figs. 10, 11) generally matched that of *Cercaria parvicaudata*. However, *F. fellis* lacked semicircular muscle fibers; the anterioradial and posterioradial muscle fibers were longer and slightly overlapped (ar and pr on Fig. 10b); and the anteriolateral muscle fibers bent sideway anteriorly (al on Figs. 10c, 11).

**Fig. 12:**
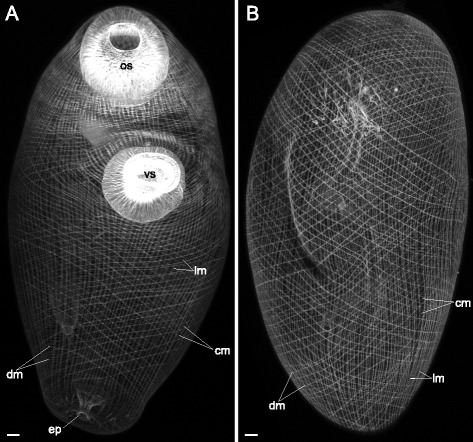
*Neophasis lageniformis* metacercariae, body-wall musculature. **a**: ventral view; **b**: dorsal view. cm – circular muscle fibers; dm – diagonal muscle fibers; ep – excretory pore; lm – longitudinal muscle fibers; os – oral sucker; vs – ventral sucker. Scale bars 10 μm

**Fig. 13:**
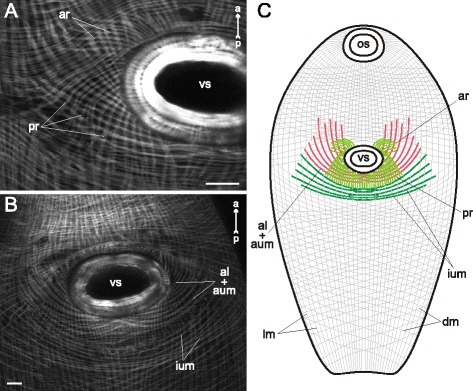
*Neophasis lageniformis* metacercariae, body-wall musculature. **a**: superficial frontal optical slice through the region of ventral sucker opening; **b**: Z-stack of frontal optical slices of midbody; **c**: scheme showing the arrangement of longitudinal, diagonal, and additional groups of muscles on the ventral side of the trunk. al + aum – anteriolateral muscle fibers with aU-shaped muscle set; ar – anterioradial muscle fibers; dm – diagonal muscle fibers; gp – genital pore; ium – muscle fibers of iU-shaped set; lm – longitudinal muscle fibers; pr – posterioradial muscle fibers; vs – ventral sucker. Scale bars 10 μm

The metacercariae of *Neophasis lageniformis* (Figs. 12, 13) had three main muscle layers well developed. The diagonal and longitudinal muscle fibers were most densely spaced and thick on the ventral side of the preacetabular region. The dorsal and the ventral sets of the diagonal muscle fibers were separate. The short and rather thick anterioradial muscle fibers were strongly bent sideway (ar on Fig. 13a, c). The longer and thinner posterioradial muscle fibers were present as well (pr on Fig. 13a, c). The anteriolateral muscle fibers were joined into thick bundles and formed the aU-shaped muscle set (al + aum on Fig. 13b, c). A wide arch of the iU-shaped muscle set was composed of thick muscle bundles (ium on Fig. 13b, c).

**Fig. 14:**
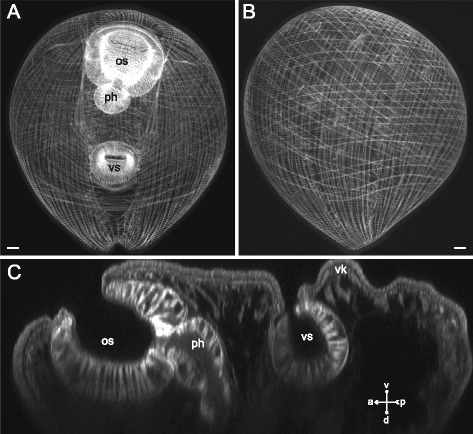
*Gymnophallus* sp. metacercariae, body-wall musculature. **a**: ventral view; **b**: dorsal view; **c**: reconstruction of middle sagittal optical slice. os – oral sucker; ph – pharynx; vk – ventral knob; vs – ventral sucker. Scale bars 10 μm

**Fig. 15:**
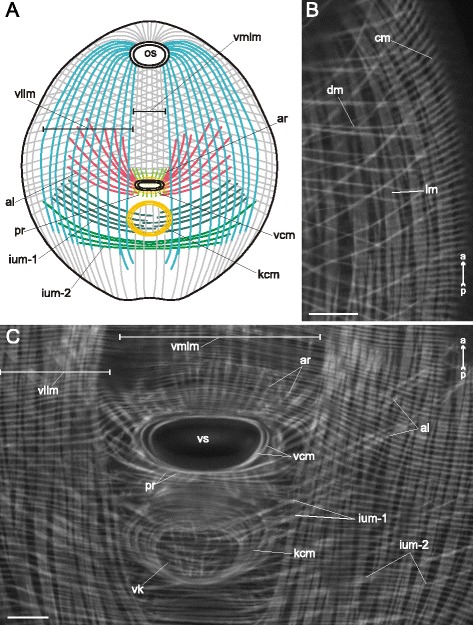
*Gymnophallus* sp. metacercariae, body-wall musculature. **a**: scheme showing the arrangement of longitudinal, diagonal, and additional groups of muscle fibers on the ventral side of the trunk; **b**: superficial frontal optical slice through the body wall (ventral); **c**: Z-stack of frontal optical slices of midbody. al – anteriolateral muscle fibers; ar – anterioradial muscle fibers; cm – circular muscle fibers; dm – diagonal muscle fibers; ium-1 and ium-2 – muscle fibers of iU-shaped sets; kcm – muscular ring around the ventral knob; lm – longitudinal muscle fibers; os – oral sucker; pr – posterioradial muscle fibers; vcm – muscular ring around the ventral sucker opening; vk – ventral knob; vllm – ventrolateral longitudinal muscle bands; vmlm – ventromedial longitudinal muscle fibers; vs – ventral sucker. Scale bars 10 μm

The plump metacercariae of *Gymnophallus* sp. (Figs. 14, 15) apart from common features possessed a ventral knob in the postacetabular region (Figs. 14c, 15c). The circular muscle fibers were closely and regularly arranged along the entire trunk of the metacercariae. The longitudinal muscle fibers formed bundles (Fig. 15b), and the most densely packed bundles were observed in the ventrolateral areas of the preacetabular region (vllm on Fig. 15a, c) whereas in the postacetabular region they rarefied and became thinner (Fig. 14a, b). The diagonal muscle fibers of the dorsal side rarefied towards the posterior end (Fig. 14b). On the ventral side they were absent in the whole postacetabular region (Fig. 14a). Seven additional groups of muscle fibers were found within the body wall of *Gymnophallus* sp. metacercariae. The anterioradial and posterioradial muscle fibers were sparse and short (ar and pr on Fig. 15a, c). Thick long bundles of the anteriolateral muscle fibers did not form the aU-shaped set (al on Fig. 15a, c). In the postacetabular region two separate iU-shaped sets of muscle bundles were found (ium-1 and ium-2 on Fig. 15a, c). Besides there were two rings of the muscle fibers: a loose one surrounding the ventral sucker, and a dense ring surrounding the ventral knob (vcm and kcm on Fig. 15a, c).

**Fig. 16:**
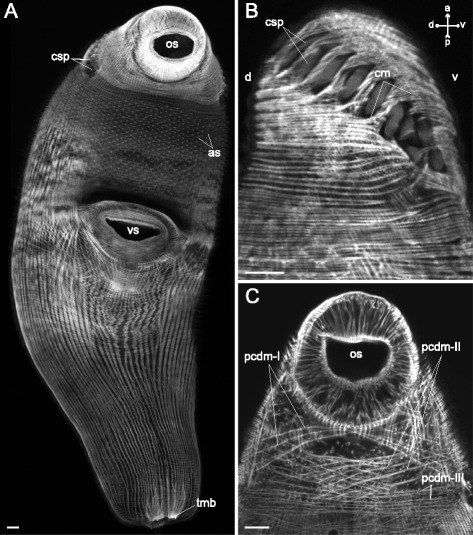
*Himasthla elongata* cercariae, body-wall musculature. **a**: trunk ventral view (tail detached); **b**: side view of the anterior region; **c**: frontal optical slice through the precollar region (close to the ventral surface). as – actinous spines in tegument; cm – circular muscle fibers; csp – collar spines; pcdm(I, II, III) – specific groups of the diagonal muscle fibers in the precollar region; os – oral sucker; tmb – dense muscle bundles close to the tail basis; vs – ventral sucker. Scale bars 10 μm

**Fig. 17:**
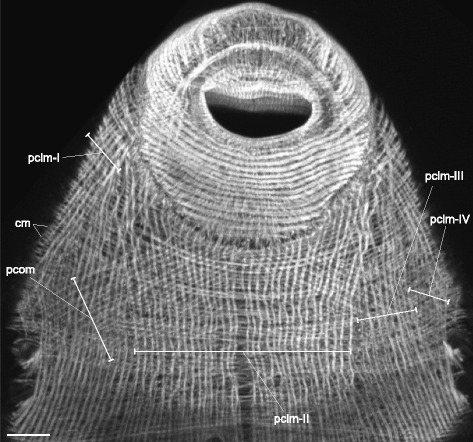
*Himasthla elongata* cercariae, body-wall musculature of the precollar region (ventral side). cm – circular muscle fibers; pclm (I to IV) – specific groups of the longitudinal muscle fibers in the precollar region; pcom – oblique muscle fibers in the precollar region. Scale bars 10 μm

**Fig. 18:**
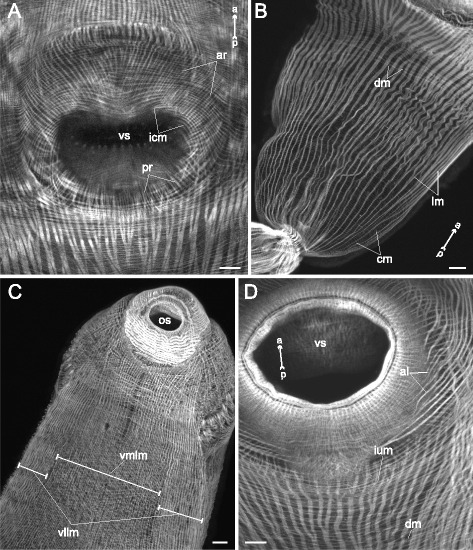
*Himasthla elongata* cercariae, body-wall musculature (ventral side). **a**: arrangement of superficial body-wall musculature around the ventral sucker opening; **b**: postacetabular region; **c**: preacetabular region; **d**: body-wall musculature near the ventral sucker. al – anteriolateral muscle fibers; ar – anterioradial muscle fibers; cm – circular muscle fibers; dm – diagonal muscle fibers; icm – bent and medially interrupted circular muscle fibers; ium – muscle fibers of iU-shaped set; lm – longitudinal muscle fibers; pr – posterioradial muscle fibers; os – oral sucker; vllm – ventrolateral longitudinal muscle bands; vmlm – ventromedial longitudinal muscle fibers; vs – ventral sucker. Scale bars 10 μm

**Fig. 19:**
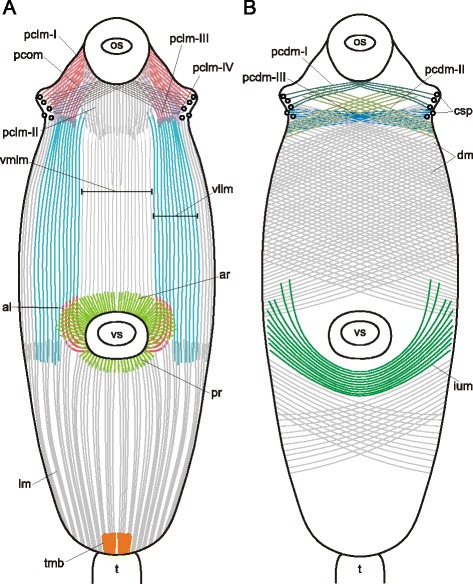
*Himasthla elongata* cercariae, schemes of the body-wall musculature (ventral side). **a**: scheme showing the arrangement of longitudinal and some additional groups of muscles; **b**: scheme showing the arrangement of diagonal and some additional muscle groups. al – anteriolateral muscle fibers; ar – anterioradial muscle fibers; csp – collar spines; dm – diagonal muscle fibers; ium – muscle fibers of iU-shaped set; lm – longitudinal muscle fibers; os – oral sucker; pcdm(I, II, III) – specific groups of the diagonal muscle fibers in the precollar region; pclm (I to IV) – specific groups of the longitudinal muscle fibers in the precollar region; pcom – oblique muscle fibers in the precollar region; pr – posterioradial muscle fibers; t – tail; tmb – dense muscle bundles close to the tail basis; vllm – ventrolateral longitudinal muscle bands; vmlm – ventromedial longitudinal muscle fibers; vs – ventral sucker. Scale bars 10 μm

**Fig. 20:**
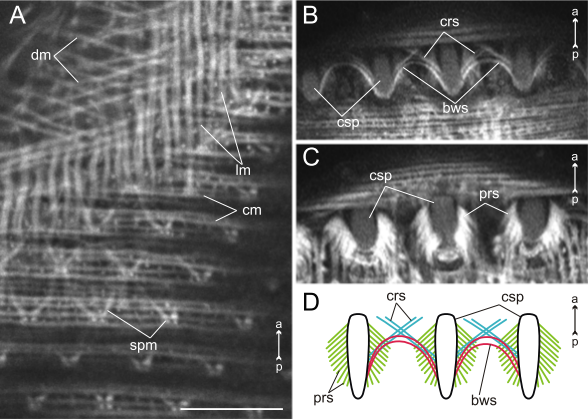
*Himasthla elongata* cercariae, spine musculature. **a**: oblique optical slice through the body wall (ventral side, preacetabular region); **b**: superficial frontal optical section through middorsal collar spines; **c**: deeper frontal optical section through middorsal collar spines; **d**: scheme showing the arrangement of muscle fibers connected with collar spines. bws – bow-shaped muscle fibers of the collar spines; cm – circular muscle fibers; crs – criss-cross muscle fibers of the collar spines; csp – collar spines; dm – diagonal muscle fibers; lm – longitudinal muscle fibers; prs – protractors of the collar spines; spm – muscle fibers connected with tegumental spines. Scale bars 10 μm

The most sophisticated musculature organization was found in *Himasthla elongata* cercariae (Figs. 16, 17, 18, 19, 20). They possess a so-called collar with large actinous spines on it. Thus the precollar region is demarcated, and we observed differentiation of its musculature. The circular fibers in the precollar region were joined into bundles, whereas along the rest of the trunk they lay separately (Figs. 16b, 17, 18b). Also they were interrupted due to the oblique position of the collar (Fig. 16b). The diagonal muscle fibers formed three distinct groups in the precollar region on the ventral side (pcdm-I, −II, −III on Figs. 16c, 19b). These groups were different in their angle of intersection. The longitudinal muscle fibers of the precollar region did not continue into the preacetabular region, but formed a separate group which could be subdivided into four clusters of different orientation (pclm-I, −II, −III, −IV on Figs. 17, 19a). Along the ventral border of the precollar region (where the collar is interrupted) these fibers interdigitated with the longitudinal muscle fibers of the preacetabular region. An additional group of oblique muscle fibers lay in the precollar region between the layers of circular and longitudinal muscle fibers (pcom on Figs. 17, 19a).

The arrangement of three main muscle layers in *Himasthla elongata* сercariae differed between the preacetabular and the postacetabular regions as well. The circular fibers did not form bundles in either of them, but in the postacetabular region they were more widely spaced. In the area lateral and anterior to the ventral sucker opening they were bent following the sucker outline, and some of them were interrupted medially (icm on Fig. 18a). The longitudinal muscle fibers were joined into bundles which were larger and wider spaced in the postacetabular region (Fig. 18b, c). Close to the tail basis the longitudinal fibers formed dense short bundles (tmb on Fig. 16a). Quite compact arrangement of the longitudinal muscle bundles was observed in the ventrolateral areas of the preacetabular region (vllm on Fig. 18c, 19a).

Four additional groups of muscle fibers were found within the body wall of *Himasthla elongata* close to the ventral sucker opening. These are long and thin anterioradial fibers, shorter and thicker posterioradial fibers (ar and pr on Figs. 18a, 19a), paired fans of the anteriolateral fibers (not forming the aU-shaped set) (al on Figs. 18d, 19a), and wide bow-shaped muscle band – the iU-shaped set (ium on Figs. 18d, 19b).

*Himasthla elongata* was the only species to demonstrate the own musculature of the tegumental spines. The common tegumental spines were chequerwise scattered throughout the preacetabular region, and each of them was connected to four muscle fibers: a pair directed anteriorly and aside, and a pair directed inward the body (spm on Fig. 20a). The musculature of the collar spines was much more advanced: the bow-shaped and criss-cross muscle fibers, and the powerful protractors (bws, crs and prs on Fig. 20b, c, d).

The total list of the body-wall muscle layers and groups for each species, and their relative position is shown in the Table 2.

**Table 2:** Musculature of the body wall

Species	Stage	Layers and groups of muscle fibers					
*Cotylurus cornutus*	Cerc	cm		lm	al	dm	
				ar	+aum		
*Saguinicola* sp.	Cerc	cm		lm		dm	
*Fellodistomum fellis*	Cerc	cm	ar	lm		dm	al
			pr			ium	
*Gymnophallus* sp.	Mc	cm	ar	lm	al	dm	
		vcm	pr	kcm		ium-1	
						ium-2	
*Neophasis lageniformis*	Mc	cm	ar	lm	al	dm	
			pr		+aum	ium	
*Himasthla elongata*	Cerc	cm	ar	lm	al	dm	
			pr				
			pcom			ium	
*Cryptocotyle lingua*	Cerc	cm		lm		dm	
*Cercaria parvicaudata*	Cerc	cm	ar	lm	al	dm	ium
		scm	pr				
*Cercaria edgesii*	Cerc	cm	ar	lm	al	dm	
					+aum	ium	
*Microphallus claviformis*	Cerc	cm		lm		dm	

Layers are ordered from left to right starting with the outmost. Cerc – cercariae; Mc – metacercariae. al – anteriolateral muscle fibers; ar – anterioradial muscle fibers; aum – U-shaped group of muscle fibers within the group of anteriolateral muscle fibers; cm – circular muscle fibers; dm – diagonal muscle fibers; ium – U-shaped group of muscle fibers separate from the anteriolateral fibers; kcm – ring of muscle fibers surrounding the ventral knob; lm – longitudinal muscle fibers; pcom – oblique muscle fibers of the precollar region; pr – posterioradial muscle fibers; scm – semicircular muscle fibers; vcm – ring of muscle fibers surrounding the ventral sucker opening

### Internal musculature

**Fig. 21:**
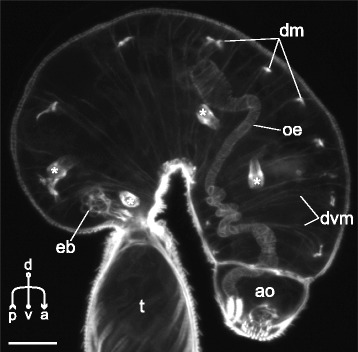
*Sanguinicola* sp. cercaria, Z-stack of sagittal optical sections showing internal muscle fibers. Asterisks show the flame cells. ao – anterior organ; dm – diagonal muscle fibers; dvm – dorsoventral muscle fibers; eb – excretory bladder; oe – esophagus; t – tail. Scale bars 10 μm

The dorsoventral muscle fibers were present in all of the examined species. In *Sanguinicola* sp. these were extremely weak and represented the only component of the internal musculature (dvm on Fig. 21 and thereafter).

**Fig. 22:**
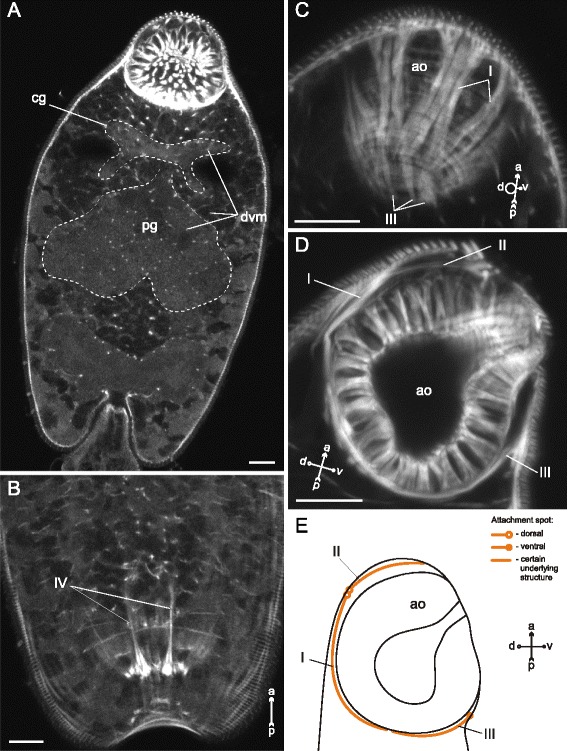
*Cryptocotyle lingua* cercariae, internal musculature. **a**: frontal optical slice through the trunk; **b**: Z-stack of frontal optical slices through the hind part of the trunk **c**: Z-stack of oblique optical sections close to the posteriodorsal surface of the anterior organ; **d**: sagittal optical section through the anterior organ; **e**: scheme of sagittal optical section through the anterior organ showing its protractors. Roman numerals mark the additional internal muscle bundles. ao – anterior organ; cg – ganglion; dvm – dorsoventral muscle fibers; pg – penetration glands. Scale bars 10 μm

In *Cryptocotyle lingua* dorsoventral muscle fibers were more numerous in the forebody than in the hindbody. Remarcably, they passed through the cerebral ganglion and between the unicellular penetration glands (Fig. 22a). Besides, the cercariae had three groups of muscle bands protracting the anterior organ (I, II, III on Fig. 22c, d, e), and a pair of longitudinal muscle bundles passing through the trunk from the ventro-genital sac primordium to the tail basis (IV on Fig. 22b).

**Fig. 23:**
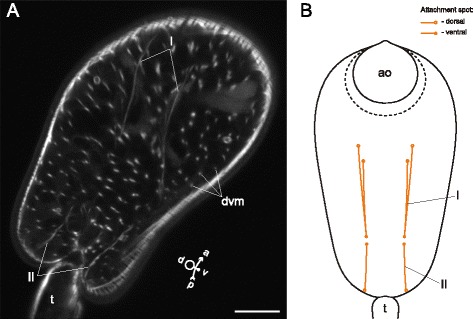
*Microphallus claviformis* cercariae, internal musculature. **a**: oblique longitudinal optical slice through the trunk; **b**: scheme illustrating the arrangement of additonal internal muscle bundles. Roman numerals mark the additional internal muscle bundles. ao – anterior organ; dvm – dorsoventral muscle fibers; t – tail. Scale bars 10 μm

*Microphallus claviformis* cercariae had dorsoventral muscle fibers uniformly arranged within the trunk. Also cercariae had two pairs of interior longitudinal muscle bundles (Fig. 23a, b).

**Fig. 24:**
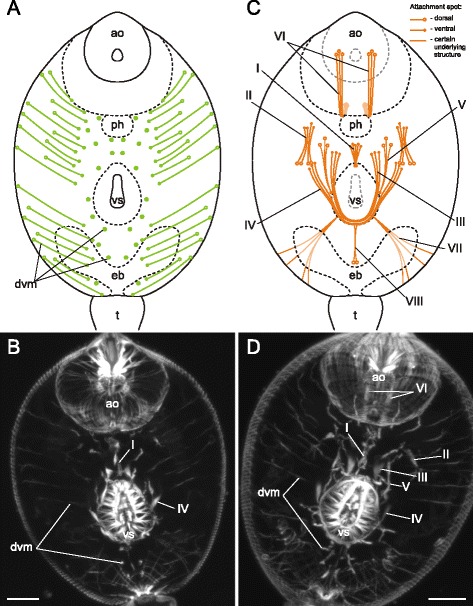
*Cercaria edgesii* cercariae, internal musculature. **a**: scheme illustrating the arrangement of dorsoventral muscle fibers; **b**: frontal optical slice of the trunk; **c**: scheme illustrating the arrangement of additonal internal muscle bundles (dorsal view); **d**: Z-stack of frontal optical slices. Roman numerals mark the additional internal muscle bundles. ao – anterior organ; dvm – dorsoventral muscle fibers; eb – excretory bladder; ph – pharynx; t – tail; vs – ventral sucker. Scale bars 10 μm

**Fig. 25:**
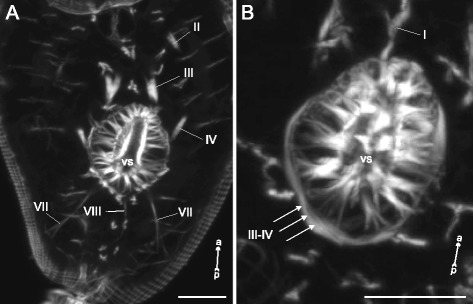
*Cercaria edgesii* cercariae, internal musculature. **a**: Z-stack of frontal optical slices of the trunk; **b**: Z-stack of frontal optical slices in the region of the ventral sucker. Roman numerals mark the additional internal muscle bundles. vs – ventral sucker. Scale bars 10 μm

The dorsoventral muscle fibers in *Cercaria edgesii* had prominent incline in the lateral regions: their dorsal ends terminated more laterally and anteriorly than the ventral ones (Fig. 24a, b). The additional interior musculature of *C. edgesii* was quite diverse and included eight groups of muscle bundles most of which were somehow connected with the anterior organ and the ventral sucker (Figs. 24b, c, d, 25). Two of these groups (III and IV on figures) formed the third U-shaped muscle set associated with the ventral sucker (Figs. 24c, d, 25b).

**Fig. 26:**
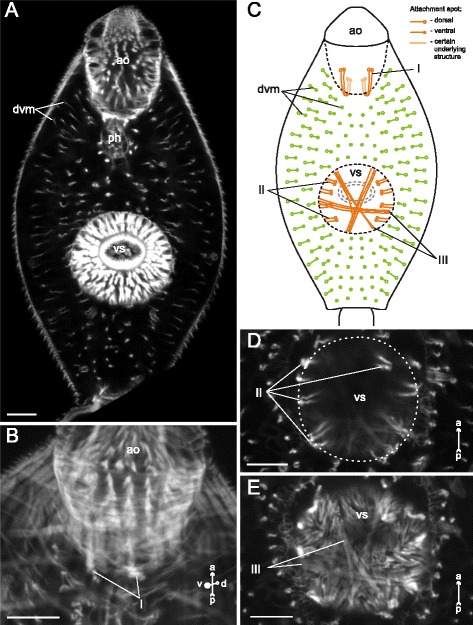
*Cotylurus cornutus* cercariae, internal musculature. **a**: frontal optical slice through the trunk; **b**: Z-stack of oblique longitudinal optical slices in the region of anterior organ; **c**: scheme illustrating the arrangement of dorsoventral and additonal internal musculature (dorsal view); **d**: Z-stack of frontal optical slices, dorsally to the ventral sucker; **e**: Z-stack of few frontal optical slices close to the dorsal surface of the ventral sucker. Roman numerals mark the additional internal muscle bundles. ao – anterior organ; dvm – dorsoventral muscle fibers; ph – pharynx; vs – ventral sucker. Scale bars 10 μm

In *Cotylurus cornutus* cercariae the dorsoventral muscle fibers were evenly arranged and demonstrated moderate incline in the lateral regions (Fig. 26a, c). Three groups of additional interior muscle bundles were observed: the anterior-organ protractors (I on Fig. 26b, c), the ventral-sucker dilators-retractors (II on Fig. 26c, d), and the ventral sucker dilators (III on Fig. 26c, e).

**Fig. 27:**
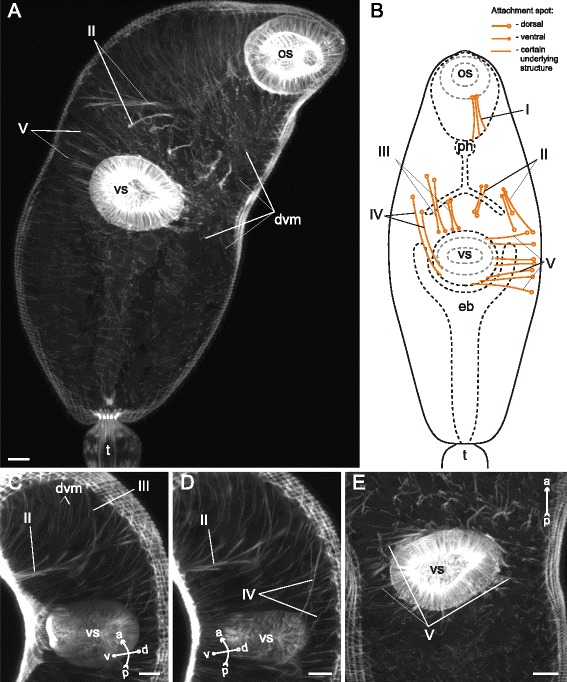
*Cercaria parvicaudata* cercariae, internal musculature. **a**: Z-stack of frontal optical slices through the trunk; **b**: scheme illustrating the arrangement of additonal internal muscles, bilaterally symmetrical groups are shown only on one side; **c**: Z-stack of sagittal optical slices in the preacetabular region; **d**: the same, more lateral slices; **e**: Z-stack of frontal optical slices in the region of ventral sucker. Roman numerals mark the additional internal muscle bundles. dvm – dorsoventral muscle fibers; eb – excretory bladder; os – oral sucker; ph – pharynx; t – tail; vs – ventral sucker. Scale bars 10 μm

In *Cercaria parvicaudata* the dorsoventral muscle fibers were again slightly inclined, and also they were much better developed in the preacetabular region than in the postacetabular one (Fig. 27a). Besides there were five groups of additional internal muscle bundles (Fig. 27), with oblique longitudinal bundles being the most conspicuous group (II on the Figure).

**Fig. 28:**
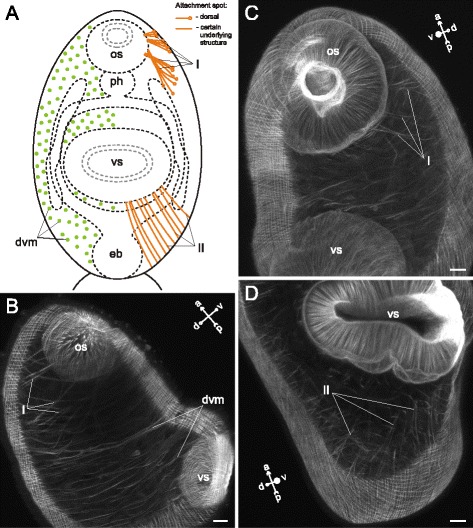
*Fellodistomum fellis* cercariae, internal musculature. **a**: scheme illustrating the arrangement of additonal internal muscle bundles (left side) and dorsoventral muscle fibers (right side); **b**: Z-stack of sagittal optical slices through the preacetabular region; **c**: Z-stack of oblique longitudinal optical slices through the preacetabular region; **d**: Z-stack of oblique longitudinal optical slices through the postacetabular region. Roman numerals mark the additional internal muscle bundles. dvm – dorsoventral muscle fibers; eb – excretory bladder; os – oral sucker; ph – pharynx; vs – ventral sucker. Scale bars 10 μm

The cercariae of *Fellodistomum fellis* had uniformly distributed dorsoventral muscle fibers (Fig. 28a, b). The additional internal muscle bundles included two groups: rather weak posterior protractors of the ventral sucker (II on Fig. 28a, d) and four bundles of oral sucker retractors (I on Fig. 28a-c).

**Fig. 29:**
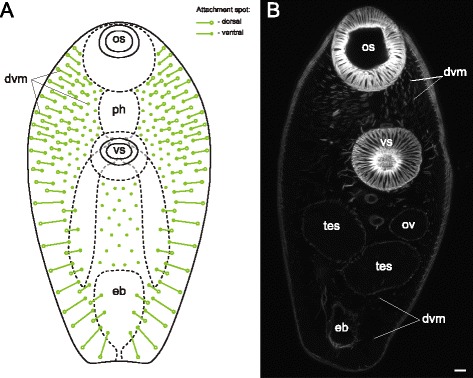
*Neophasis lageniformis* metacercariae, dorsoventral muscle fibers. **a**: scheme illustrating the arrangement of dorsoventral muscle fibers; **b**: Z-stack of few frontal optical slices through the body. c – caecum; dvm – dorsoventral muscle fibers; eb – excretory bladder; os – oral sucker; ov – ovary; ph – pharynx; tes – testis; vs – ventral sucker. Scale bar 10 μm

**Fig. 30:**
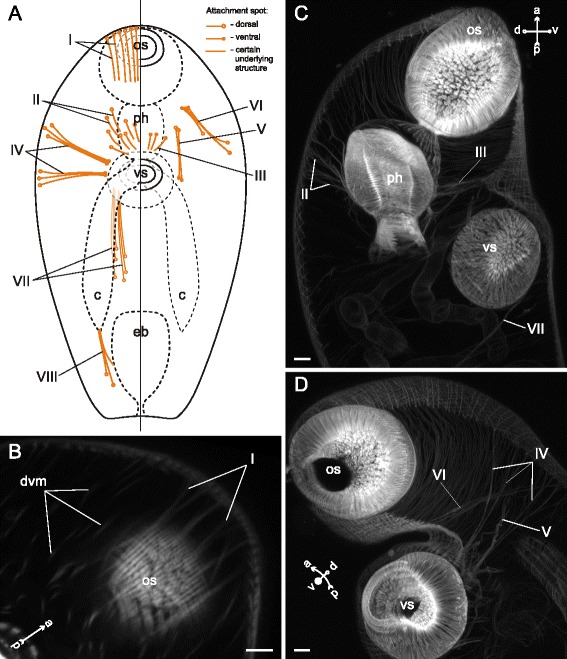
*Neophasis lageniformis* metacercariae, internal musculature. **a**: scheme illustrating the arrangement of additonal internal muscle bundles (left – dorsal view; right – ventral view), bilaterally symmetrical groups are shown only on one side; **b**: Z-stack of few frontal optical slices close to the dorsal surface of the oral sucker; **c**: Z-stack of sagittal optical slices of the preacetabular region; **d**: Z-stack of oblique optical slices of the preacetabular region. Roman numerals mark the additional internal muscle bundles. c – caecum; dvm – dorsoventral muscle fibers; eb – excretory bladder; os – oral sucker; ph – pharynx; vs – ventral sucker. Scale bars 10 μm

**Fig. 31:**
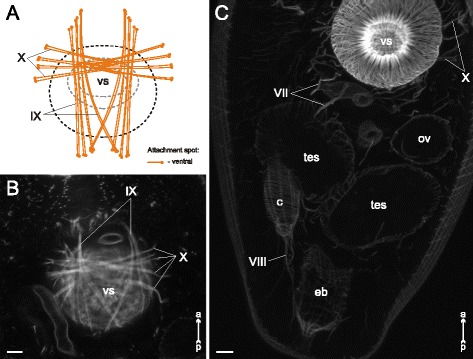
*Neophasis lageniformis* metacercariae, internal musculature. **a**: scheme illustrating the arrangement of ventral sucker protractors (IX and X) additonal internal muscle bundles; **b**: Z-stack of few frontal optical slices close to the dorsal surface of the ventral sucker; **c**: Z-stack of frontal optical slices of the postacetabular region. Roman numerals mark the additional internal muscle bundles. c – caecum; eb – excretory bladder; ov – ovary; tes – testis; vs – ventral sucker. Scale bars 10 μm

In *Neophasis lageniformis* metacercariae the dorsoventral muscle fibers were more densely arranged in the preacetabular region (Fig. 29a, c, d). In both regions most of them were inclined: their dorsal ends terminated further from the center of the trunk than the ventral ones. There were ten additional groups of the internal muscle fibers (Figs. 30, 31), and the most dense among them were the retractors of the pharynx (II and III on Fig. 30a, c)

**Fig. 32:**
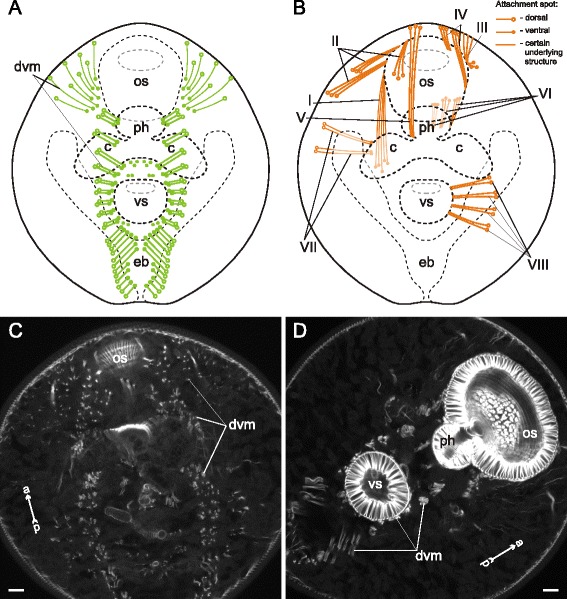
*Gymnophallus* sp. metacercariae, internal musculature. **a**: scheme illustrating the arrangement of dorsoventral muscle fibers; **b**: scheme illustrating the arrangement of additonal internal muscle bundles (dorsal view), bilaterally symmetrical groups are shown only on one side; **c**: frontal optical slice of the trunk closer to the dorsal surface; **d**: frontal optical slice of the trunk closer to the ventral surface. Roman numerals mark the additional internal muscle bundles. c – caecum; dvm – dorsoventral muscle fibers; eb – excretory bladder; os – oral sucker; ph – pharynx; vs – ventral sucker

**Fig. 33:**
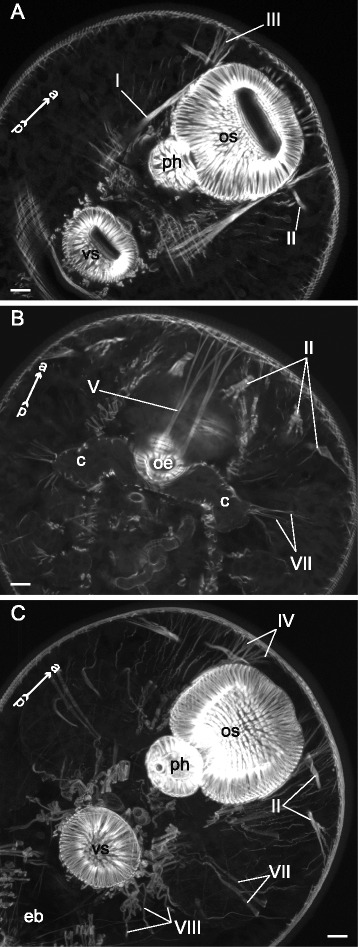
*Gymnophallus* sp. metacercariae, internal musculature. **a**: Z-stack of few frontal optical slices close to the ventral surface; **b**: Z-stack of few frontal optical slices close to the dorsal surface; **c**: Z-stack of frontal optical slices. Roman numerals mark the additional internal muscle bundles. c – caecum; eb – excretory bladder; oe – esophagus; os – oral sucker; ph – pharynx; vs – ventral sucker. Scale bars 10 μm

The dorsoventral muscle fibers of *Gymnophallus* sp. metacercariae were compactly arranged in two longitudinal rows and clearly inclined (Fig. 32a, c, d). The metacercariae also possessed eight groups of additional internal muscle bundles (Fig. 32b). All of them occurred in the preacetabular region. The most prominent among them were the oral sucker retractors (I and II on Figs. 32b, 33) and protractors (IV on Figs. 32b, 33).

**Fig. 34:**
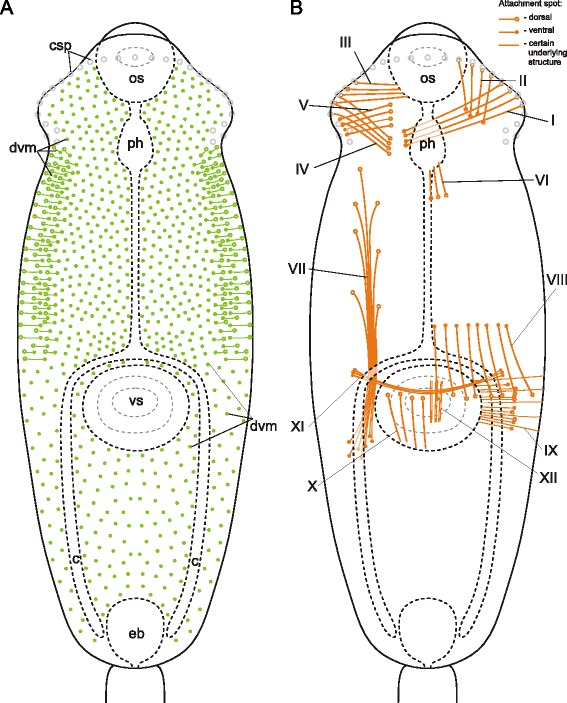
*Himasthla elongata* cercariae, internal musculature. **a**: scheme illustrating the arrangement of dorsoventral muscle fibers; **b**: scheme illustrating the arrangement of additonal internal musculature (dorsal view), bilaterally symmetrical groups are shown only on one side. Roman numerals mark the additional internal muscle bundles. c – caecum; csp – collar spines; dvm – dorsoventral muscle fibers; eb – excretory bladder; os – oral sucker; ph – pharynx; vs – ventral sucker. Scale bars 10 μm

**Fig. 35:**
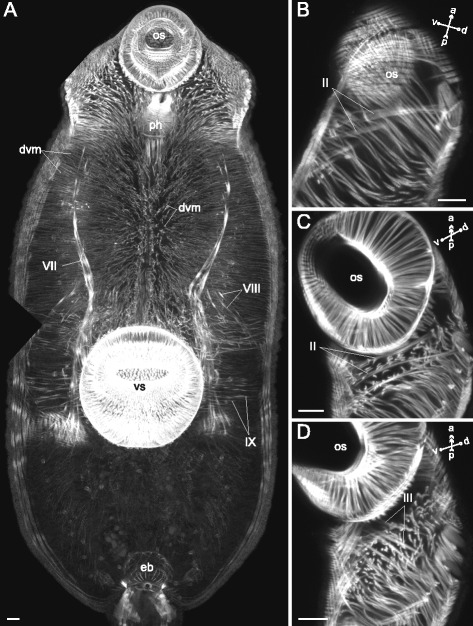
*Himasthla elongata* cercariae, internal musculature. **a**: Z-stack of frontal optical slices of the whole trunk; **b**: Z-stack of sagittal optical slices of the precollar region; **c**: Z-stack of oblique optical slices of the precollar region; **d**: the same, slices close to the surface. Roman numerals mark the additional internal muscle bundles. dvm – dorsoventral muscle fibers; eb – excretory bladder; os – oral sucker; ph – pharynx; vs – ventral sucker. Scale bars 10 μm

**Fig. 36:**
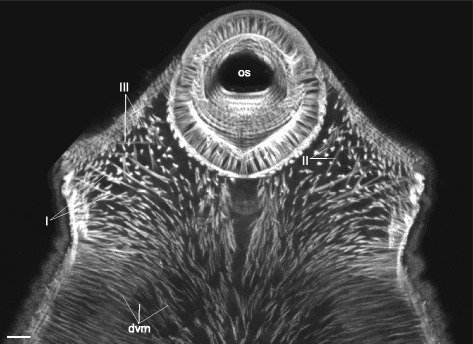
*Himasthla elongata* cercariae, internal musculature in the precollar region. Z-stack of frontal optical slices. Roman numerals mark the additional internal muscle bundles. dvm – dorsoventral muscle fibers; os – oral sucker. Scale bar 10 μm

**Fig. 37:**
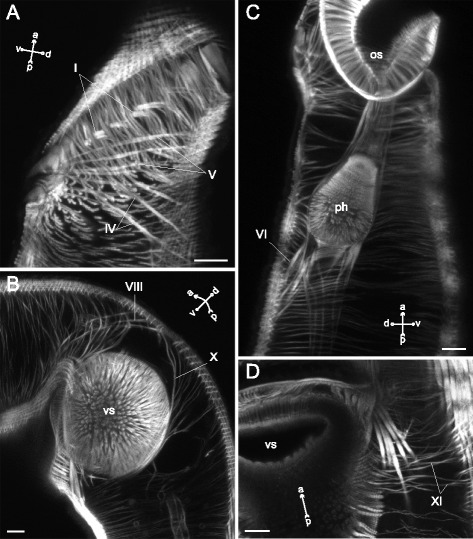
*Himasthla elongata* cercariae, internal musculature. **a**: Z-stack of sagittal optical slices of the anterior region (collar and the anterior part of preacetabular region); **b**: Z-stack of sagittal optical slices of the midbody; **c**: Z-stack of sagittal optical slices in the preacetabular region; **d**: Z-stack of frontal optical slices near the ventral sucker. Roman numerals mark the additional internal muscle bundles. os – oral sucker; ph – pharynx; vs – ventral sucker. Scale bars 10 μm

*Himasthla elongata* cercariae had the dorsoventral muscle fibers much better developed in the preacetabular region, and these fibers were strongly inclined (Figs. 34a, 35a, 36). The additional internal musculature included twelve groups of muscle fibers, most of them in bundles (Fig. 34b). Five of these groups in the precollar region were connected with the collar spines (I to V on Figs. 34b, 35b-d, 36, 37a). All of the other groups were located in the preacetabular region (Figs. 34b, 35a, 37b, d). The longitudinal muscle bundles were the largest (VII on Figs. 34b, 35a).

## Discussion

### Body-wall musculature

The presence of three main muscle layers (outer circular, intermediate longitudinal and inner diagonal) within the body wall is typical for the trematode hermaphroditic generation [16, 17, 31]. The alteration of this scheme is rare and appears due to deep specialization within single taxa, e.g. the layer of diagonal muscle fibers is substituted by the second layer of circular muscle fibers in the hindbody of Strigeidea [24]; an additional inner longitudinal layer is present in Paramphistomata [32, 33]; etc. However, among our material only highly juvenilized cercariae without ventral sucker had three muscle layers of the body wall exclusively. The rest possessed a number of additional groups of muscle fibers, and the most common among them were anterioradial, posterioradial, anteriolateral muscle fibers, and U-shaped muscle sets. Another frequent modification was the enhancement of the longitudinal muscle fibers in ventrolateral areas: as a result the ventrolateral longitudinal bands formed. Note that features listed above were common in the species having the ventral sucker and hence the primary differentiated trunk. All the main additional muscle groups were somehow associated with the ventral sucker. This makes us suppose that they enhance the agility of the preacetabular region, e.g. leech-like locomotion and movements during the second intermediate host infection when the cercaria attaches to the host by the ventral sucker and penetrates the host epithelium with the anterior organ. On the contrary the body-wall musculature in the postacetabular region is rarefied. Such a morphological distinction between the two regions supports the hypothesis of the trunk functional differentiation [18]. In previously studied species the musculature differentiation between two trunk regions is evident in schistosomatid cercariae [26, 34] and in *Echinostoma caproni* [20].

The common additional body-wall muscle groups were probably acquired later in evolution than three main muscle layers. Since these structures were found in species from distant taxa, they cannot be regarded as a result of narrow specialization. Thus we consider the listed muscle groups to be peculiar characteristics of the trematode hermaphroditic generation musculature. Here we presume that this pattern is characteristic for any stage (cercariae, metacercariae and adult worms) which has primarily differentiated trunk. Quite often the morphogenesis of hermaphroditic generation goes gradually (except for the larval provisional organs), so that the primary trunk differentiation is preserved from cercariae to adult [16]. However this is not the case for *Cotylurus cornutus* and any other Diplostomoidea, as they have complex metamorphosis of cercaria into metacercariae. In the course of such metamorphosis significant transformation of musculature was described recently in *Diplostomum pseudospathaceum* [35].

The presence of the anteriolateral fibers, U-shaped muscle sets and ventrolateral longitudinal bands leads to another important consequence – formation of an annular structure on the ventral surface in the preacetabular region. The U-shaped sets of muscle fibers and the ventral sucker form the posterior confine, the ventrolateral longitudinal bands form the lateral boundaries, and the oral sucker (or the anterior organ) constrains the area anteriorly. The ventrolateral longitudinal bands are linked to the posterior confine by the anteriolateral muscle fibers and/or the lateral parts of the iU-shaped muscle set. Thus the annular structure integrates the oral sucker (or the anterior organ) and the ventral sucker. A curious fact is that several acoelomorph flatworms are known to possess resembling structures. These are usually formed by the cross-over muscle fibers (e.g. in *Convoluta pulchra* [5], *Haplogonaria phyllospadicis* [36], *Convolutriloba longifissura* [7]) and the U-shaped muscle sets (e.g. *Eumecynostomum asterium*, *Pseudaphanostoma smithrii* [6]). Also the annular muscle structure may function as an outline of the ventral concavity occupying the preacetabular region (see below).

### Internal musculature

The dorsoventral muscle fibers are abundant in both parasitic and free-living flatworms, and are thought to maintain the flattened body shape [21, 37–39]. We should point out two specific features in the arrangement of the dorsoventral muscle fibers. The first is the incline of the dorsoventral muscle fibers in such way that their dorsal ends are attached further from the center of the trunk than the ventral ones. This was found in seven species. We may expect the inclined dorsoventral fibers to create tension when the trunk is constantly curved on the ventral side. This is observed, for instance, in swimming cercaria – it obviously helps to reduce the resistance of water. The second character was distinct in four species: the array of dorsoventral fibers in the preacetabular region was denser than in the postacetabular one. This again supports the differentiation of the preacetabular region towards the locomotory function. Also, the arrangement of dorsoventral muscle fibers indicates the possibility that the whole ventral surface of the trunk, or at least the preacetabular region, serves for attachment. Such a phenomenon is known for Notocotylidae as the adhesion by the ventral concavity. The negative pressure in this concavity is formed like in a sucker, and the dorsoventral muscle fibers act in this case like the radial muscle fibers of the sucker [17, 40].

Eight main types of the additional internal musculature were defined on the basis of functional and/or morphological affinity: (1) the oral sucker or the anterior organ protractors, (2) the oral sucker retractors, (3) the ventral sucker protractors and/or dilators, (4) the ventral sucker retractors, (5) the transverse dilators-retractors of the ventral sucker, (6) the transverse muscle bundles of the preacetabular region, (7) the criss-cross groups of muscle bundles, and (8) the retractors of the pharynx (Table 3). The group of ventral sucker protractors and/or dilators actually may be divided in two: longitudinal and transverse bands. Most of all these groups are somehow connected with the suckers or the anterior organ, and probably manage movements of these organs relative to the trunk. We suppose that the transverse and criss-cross muscle groups are used to support tension when the trunk is ventrally curved, together with the dorsoventral muscle fibers and musculature of the body wall.

**Table 3:** Main types of the additional internal muscle fibers and bundles

Species	Stage	Types of additional internal muscle fibers and bundles are labeled with arabic numerals according to the list in results section.							
		1	2	3	4	5	6	7	8
*Cotylurus cornutus*	Cerc	I		III		II			
*Fellodistomum fellis*	Cerc		I		II				
*Gymnophallus* sp.	Mc	IV	I, II			VIII	VII		IV, V
*Neophasis lageniformis*	Mc	I		IX, X	VII		IV	V,VI	II, III
*Himasthla elongata*	Cerc			XI, XII	X	IX		VII, VIII	VI
*Cryptocotyle lingua*	Cerc	I, II, III							
*Cercaria parvicaudata*	Cerc	I			IV	V		II, III	
*Cercaria edgesii*	Cerc	VI		III, IV	VII, VIII			II, V	
*Microphallus claviformis*	Cerc							I, II	

*Cerc* cercariae, *Mc* metacercariae. Roman numerals stand for the groups of additional internal musculature of certain species according to the figure lettering. 1 – oral sucker protractors; 2 – oral sucker retractors; 3 – ventral sucker protractors and/or dilators: 4 – ventral sucker retractors; 5 – transverse dilators-retractors of the ventral sucker; 6 – transverse muscle bundles in the preacetabular region; 7 – criss-cross groups of muscle bundles; 8 – retractors of the pharynx

Our classification of the internal musculature is primarily based on function. However, if we look for homologous structures, they should be similar at least in both function and morphology, particularly position (though strict homology according to Remane’s criteria cannot be stated based on our data). The retractors of the ventral sucker are not morphologicaly uniform and obviously have different origin. In contrast the morphological uniformity is significant within the oral sucker/anterior organ protractors, the oral sucker retractors, the transverse dilators-retractors of the ventral sucker, and the protractors and/or dilators of the ventral sucker. So these muscle groups may well be homologous among different species. Function of the transverse and criss-cross internal muscle bundles is speculative, and they were defined on the base of morphology, but still may be considered homologous.

Part of the additional internal musculature is likely to be derived from the dorsoventral fibers, at least the bundles which connect the dorsal and ventral sides of the trunk. However some may have different origin. For instance, the additional internal muscle bundles in the precollar region of *Himasthla elongata* probably derived from the diagonal muscle fibers of the body wall.

### Notes on evolution of flatworm muscle system

The somatic musculature organization in worm-like organisms appears to be highly variable. Nevertheless, the simplest orthogonal grid of outer circular and inner longitudinal muscle fibers (evident in Catenulidae and several Acoelomorpha [6]) is still considered to be the muscular ground pattern of Urbilateria [41, 42]. The question is: how would this plain pattern evolve along with the changes in the body construction? These include changes in shape and size, position of the mouth and other openings; presence of the appendages, axial regionalization of the body.

The increase of size and the flatterning of the body inevitably lead to the formation of diagonal and dorsoventral muscle fibers. The location of mouth opening undoubtedly affects the musculature pattern around it. For instance, within non-neodermatan Rhabditophora the species with uniform musculature pattern (*Urastoma cyprinae* and *Castrella truncata* [14, 15]) have simple body construction and terminal openings (mouth and common genital opening on the opposite ends in *Castrella truncata*, and orogenital pore on the posterior end in *Urastoma cyprinae*). On the contrary, species of *Macrostomum* with unconventional musculature patterns [4, 10] have mouth opening in the ventral, not terminal, position, and conspicuous caudal adhesive plate. The musculature pattern is also altered behind ventral mouth opening of *Melloplana ferruginea* juveniles [37]. Furthermore within the Acoelomorpha the musculature modifications are most typical for the dorsoventrally flatterned species with midventral position of the mouth opening (e.g. *Meara stichopi* [11], *Symsagittifera roscoffensis* [9], *Convoluta pulchra* [12] – versus *Paratomella* sp. [12], *Solenofilomorpha “crezeei”* [6]). The appearence of any outgrowths (e.g. lobes and oral hood in polyclad larvae) is essentially accompanied by specialization of associated musculature [43–45]. Within Neodermata the muscle system is greatly affected by the presence of the attachment organs: haptor in Monogenea [46–48] and scolex in Cestoda [49–51].

The body construction of trematode hermaphroditic generation is an infrequent case of clear axial regionalization among the flatworms. However, part of trematode taxa has derived various kinds of atypical morphology. On one hand there are forms with secondary differentiated trunk, e.g. Strigeidae, which develop quite different musculature in forebody and hindbody [24, 25]. On the other hand there are several groups with the secondary undifferentiated trunk: Paramphistomata, Notocotylidae, Eucotylidae, etc. Among them only paramphistomes muscle system was widely studied as it is applied for systematics of this group [52].

A wider research on both free-living and parasitic flatworms is required to develop the idea that body construction affects the somatic musculature organization. And the trematodes due to their remarkably variable appearance seem to be favourable to show the specialization potential of musculature within the flatworm *Bauplan*.

## Conclusions

The presence of the ventral sucker and the division of the trunk into the preacetabular and the postacetabular regions strongly affect the organization of somatic musculature in trematodes. The preacetabular region along with the ventral sucker is specialized for locomotion – leech-like crawling, movements during the infection of the second intermediate host, etc. The specialization of the preacetabular region leads to the development of both the internal and body-wall additional musculature. The anterioradial, posterioradial, and anteriolateral muscle fibers, U-shaped muscle sets, and dense ventrolateral longitudinal muscle bands are the basic additional muscle groups within the body wall. We propose that these groups should be considered as a part of musculature ground pattern in trematode hermaphroditic generation.

Our results fill the notable gaps in the knowledge on the flatworm muscle system and, moreover, show one peculiar possible direction in the flatworm musculature specialization.

## Methods

### Animals

Most of the material was collected in 2010—2013 at the White Sea (Kandalaksha Gulf, Chupa Inlet, Keret Archipelago), at the Barents Sea (water area near the rural locality Dalniye Zelentsy), and in the Leningrad Oblast, Russia. Three species (*Cotylurus cornutus*, *Sanguinicola* sp. and *Cercaria edgesii*) were collected by Sergei Shchenkov in 2012 in the Samara Oblast, Russia. The list of all studied species with indications of life-cycle stages is given in Table 1. This Table also contains information about the hosts and the number of specimens of each object used for the musculature description. Animal experimentation was carried out according to international and Russian ethics guidelines.

### Fluorescent staining and confocal miscroscopy

All the material was fixed and stored in 4 % solution of paraformaldehyde in phosphate-buffered saline (PBS). Specimens were washed in PBS with Triton-X100 (0,1 %) during 24 h before staining. Incubation in TRITC-labelled phalloidin solution (200 ng/ml) took another 24 h, followed by 2 h wash in PBS. Finally the specimens were mounted in glycerol/PBS (9/1) and examined under the confocal scanning laser microscopes (CSLM) Leica TCS-SP5 or Leica TCS-SPE.

ImageJ v. 1.46r software was used to process data from CSLM: to make snapshots and Z-stacks. The reconstructions of optionally directed optical slices were made using plugin “Volume Viewer” v. 1.31. Schemes and plate setups were done with Corel Draw 12 and appropriate image modifications were done with Adobe Photoshop CS2.
